# 

**Published:** 1894-08

**Authors:** 


### CONTRIBUTORS TO VOLUME XXXIV.

| | |
|---|---|
| Aaron, Charles D. | Detroit. |
| Angell, Edward B. | Rochester. |
| Bartlett, F. W. | Buffalo. |
| Benedict, A. L. | Buffalo. |
| Bissell, William G. | Buffalo. |
| Clark, George E. | Skaneateles. |
| Clark, Horace | Buffalo. |
| Conklin, W. L. | Rochester. |
| Cott, George F. | Buffalo. |
| Curtis, Harlow C. | Buffalo. |
| Daniels, Clayton M. | Buffalo. |
| Dorr, Samuel G. | Buffalo. |
| Doyle, Gregory | Syracuse. |
| Elsner, Henry L. | Syracuse. |
| Elsner, S. L. | Rochester. |
| Ford, Willis E. | Utica. |
| Frye, Maud J. | Buffalo. |
| Goler, George W. | Rochester. |
| Grosvenor, J. W. | Buffalo. |
| Hall, A. L. | Fair Haven. |
| Hartwig, Marcell. | Buffalo. |
| Hayd, Herman E. | Buffalo. |
| Hinkel, Frank Whitehill | Buffalo. |
| Hopkins, Henry Reed | Buffalo. |
| Howe, Lucien | Buffalo. |
| Hubbell, Alvin A. | Buffalo. |
| Kirk, William Redin | Louisville. |
| Krauss, William C. | Buffalo. |
| Lanphear, Emory | St. Louis. |
| Letchworth, William Pryor | Portageville. |
| Mann, Frederick J. | Watertown. |
| Marcus, Herman D. | Philadelphia. |
| Milliken, S. E. | New York. |
| Mitchell, S | Hornellsville. |
| Moehlau, F. G. | Buffalo. |
| Mynter, Herman | Buffalo. |
| Park, Roswell | Buffalo. |
| Pohlman, Julius | Buffalo. |
| Potter, William Warren | Buffalo. |
| Pryor, John H. | Buffalo. |
| Putnam, James Wright | Buffalo. |
| Rafter, George W. | Rochester. |
| Ring, Charles A. | Buffalo. |
| Robinson, Byron | Chicago. |
| Rosenberg, A. | Berlin. |
| Satterlee, Richard H. | Buffalo. |
| Sellstedt, Lars Gustav | Buffalo. |
| Stafford, James | New York. |
| Stockton, Charles G. | Buffalo. |
| Tait, Mr. Lawson | Birmingham. |
| Thornbury, Frank J. | Buffalo. |
| Trowbridge, Grosvenor R. | Buffalo. |
| Van de Warker, Ely | Syracuse. |
| Van Peyma, P. W. | Buffalo. |
| Wende, Ernest | Buffalo. |

Williams, H. T. . . . . . . . . . . . . . . . . . . . . . . . . Rochester.
Young, A. A. . . . . . . . . . . . . . . . . . . . . . . . . Newark, N. Y.
American Association of Obstetricians and Gynecologists.
Buffalo Academy of Medicine.
Buffalo Academy of Medicine, Surgical Section.
Eighth Ophthalmological Congress.
Louisville Surgical Society.
Medical Association of Central New York.
Medical Society of the County of Chautauqua.
Medical Society of the County of Erie.

### ILLUSTRATIONS IN VOLUME XXXIV.

Page.

Abdominal Pregnancy. (Williams.) . . . . . . . . . . . . . . . . . . . . 393
Ambulance Car. (St. Louis Health Department.) . . . . . . . . . . . . . 497
Ambulatory Splint. (Hartwig.) . . . . . . . . . . . . . . . . . . . . . 222
An Anencephalic Monster. (Angell-Elsner.) . . . . . . . . . . . . . . 463
Antitoxin in Buffalo.
Bacteriologists Inoculating a Horse . . . . . . . . . . . . . . . . . 421
Buffalo General Hospital . . . . . . . . . . . . . . . . . . . . . . . 555
Club Foot. (Doyle.)
Fig. 1.—Patient Walking, showing the feet everted by Spiral Rotator 707
Fig. 2.—Patient Sitting, showing Flexibility of Spiral Rotator . . . 707
Helmholtz, Professor . . . . . . . . . . . . . . . . . . . . . . . . . 251
Holmes, Oliver Wendell, facing . . . . . . . . . . . . . . . . . . . . 249
King Thermometer Syringe . . . . . . . . . . . . . . . . . . . . . . . 752
Nasal Stenosis. (Mitchell.) . . . . . . . . . . . . . . . . . . . . . 580
Neurological Bust. (Krauss.) . . . . . . . . . . . . . . . . . . . . . 471
Neurologists' Percussion Hammer. (Krauss.) . . . . . . . . . . . . . . 416
School Wardrobe . . . . . . . . . . . . . . . . . . . . . . . . . . . 559
Statue of J. Marion Sims, facing . . . . . . . . . . . . . . . . . . . 303

### Society Meetings.

The American Association of Obstetricians and Gynecologists will hold its seventh annual meeting at Toronto, Ont., Wednesday, Thursday and Friday, September 19, 20 and 21, 1894, to which the medical profession is cordially invited.

The following is the preliminary program, subject to amendment until September 1st, namely: (1) President's address, George H. Rohé, Catonsville, Md.; (2) Personal Experience with Pus Tubes: When to Operate, How to Operate, and the Results of Operation, Jas. F. W. Ross, Toronto, Ont.; (3) Relation of Hysteria to Structural

Changes in the Uterus and Adnexa, A. P. Clarke, Cambridge, Mass.; (4) Demonstration of a Mechanism of Intussusception (rabbits), Robert T. Morris, New York; (5) Nephrectomy, L. H. Dunning, Indianapolis; (6) Treatment of Distension of the Fallopian Tubes Without Laparatomy and Removal, Frank A. Glasgow, St. Louis; (7) Hysteria in Pregnancy, W. P. Manton, Detroit; (8) Relations of Renal Insufficiency to Operations, Carlton C. Frederick, Buffalo; (9) *a*, Importance of Recognizing Septic Puerperal Endometritis Early, and Its Treatment; *b*, Demonstration of a Portable Operating Table for Gynecological and Abdominal (Trendelenberg) Work, Edward J. Ill, Newark, N. J.; (10) Suspension of Retroflexed Uterus by the Utero-ovarian Ligaments, with Report of Cases, Reuben Peterson, Grand Rapids, Mich.; (11) The Element of Habit in Gynecic Disease, Geo. F. Hulbert, St. Louis; (12) Some Results of Ether Anesthesia in Abdominal Operations, I. S. Stone, Washington, D. C.; (13) Report in Abdominal Surgery, Presenting Cases, A. Vander Veer, Albany; (14) Supplementary Paper on Abdominal Section in Intrapelvic Hemorrhage, M. Rosenwasser, Cleveland; (15) Conservative Midwifery, J. M. Duff, Pittsburg; (16) The Cause of the Thirst Following Abdominal Section, Eugene Boise, Grand Rapids, Mich.; (17) The Care of Pregnant Women, W. B. Dewees, Salina, Kan.; (18) Subject to be announced, L. S. McMurtry, Louisville, Ky.; (19) *Discussion*—Inflammatory Disease of the Uterus and Appendages and of the Pelvic Peritoneum. (*a*) Introductory Remarks, William Warren Potter, Buffalo; (*b*) Historical Sketch, Edward J. Ill, Newark, N. J.; (*c*) Clinical History, Charles A. L. Reed, Cincinnati, O.; (*d*) Causation and Pathology, Lewis S. McMurtry, Louisville, Ky.; (*e*) Diagnosis and Prognosis, James F. W. Ross, Toronto, Can.; (*f*) Treatment, M. Rosenwasser, Cleveland, O.; A. Vander Veer, Albany, N. Y.; J. H. Carstens, Detroit, Mich.; A. H. Cordier, Kansas City, Mo.; (*g*) Results—(*a*) When Untreated; (*b*) Under Various Methods of Treatment, Joseph Price, Philadelphia, Pa.; (20) Intercurrent Typhoid Fever in Pregnancy, Thomas E. McArdle, Washington, D. C.; (21) Notes on a Case of Cholelithiasis, Frederick Blume, Allegheny, Pa.; (22) Perineal Operations, Joseph Price, Philadelphia; (23) Remarks Bearing on the Surgical Treatment of Intussusception in Infants, Based on Two Successful Cases, Henry Howitt, Guelph, Ont.; (24) The Limitations of Surgery in the Treatment of the Uterus and its Appendages, William H. Myers, Fort Wayne, Ind.; (25) The Incision in Abdominal Surgery—Methods and Results, J. H. Carstens, Detroit, Mich.; (26) Abdominal Section in Ectopic Gestation, where the Fetus is Living and Viable, X. O. Werder, Pittsburg, Pa.; (27) Subject to be announced, William E. B. Davis, Birmingham, Ala.; (28) Hysterectomy for Cancer of the Uterus, E. W. Cushing, Boston, Mass.

---

The American Publishers' Association, in consequence of the closing of the hotel at White Sulphur Springs, Va., has changed its meeting place to Hot Springs, Va., where it will convene on Monday and Tuesday, August 13 and 14, 1894, under the presidency of Dr. Landon B. Edwards, of Richmond, Va. All medical publishers are cordially invited to attend. All who contemplate attending, are requested to advise the secretary, Charles Wood Fassett, Esq., St. Joseph, Mo., in order that provision may be made for their accommodation.

---

The American Electro-Therapeutic Association will hold its fourth annual meeting at the Academy of Medicine in the city of New York, September 25, 26 and 27, 1894, under the presidency of Dr. William J. Herdman, of Ann Arbor. Members of the medical profession are cordially invited to attend.

---

The American Public Health Association will hold its twenty-second annual meeting at Montreal, Canada, September 25, 26, 27 and 28, 1894, under the presidency of Dr. E. B. Lachapelle, of Montreal. The following topics have been selected for consideration at this meeting: 1. The Pollution of Water Supplies. 2. The Disposal of Garbage and Refuse. 3. Animal Diseases and Animal Food. 4. The Nomenclature of Diseases and Forms of Statistics. 5. Protective Inoculations in Infectious Diseases. 6. National Health Legislation. 7. The Cause and Prevention of Diphtheria. 8. Causes and Prevention of Infant Mortality. 9. The Restriction and Prevention of Tuberculosis. 10. Car Sanitation. 11. The Prevention of the Spread of Yellow Fever. Upon all of the above subjects special committees have been appointed; therefore all papers upon these topics should be presented to the appropriate committee in season, to be incorporated as a part of the report of the committee, if deemed advisable.

The executive committee announces the following additional subjects upon which papers are invited: 12. On the Education of the Young in the Principles of Hygiene. 13. Private Destruction of Household Garbage and Refuse. 14. Disinfection of Dwellings after Infectious Diseases. 15. Inspection of School Children with reference to the Eyesight. Dr. Irving A. Watson, of Concord, N. H., is the secretary.

---

THE American Microscopical Society will hold its seventeenth annual meeting on Monday, Tuesday and Wednesday, August 13, 14 and 15, 1894, at the Polytechnic Institute, Brooklyn, N. Y.

---

THE American Association for the Advancement of Science will hold its next annual meeting at Brooklyn, N. Y., August 13, 14 and 15, 1894.

---

THE Medical Association of Central and Western New York will hold its annual meeting in Buffalo, Tuesday, October 2, 1894. A change of date has been made on account of the meetings of many of the national medical societies during the summer months.

---

THE Lehigh Valley Medical Association will hold its fourteenth annual meeting at the Hotel Penn, Reading, Pa., Thursday, August 9, 1894. The annual address before the association will be delivered by Dr. J. C. Wilson, professor of medicine in Jefferson Medical College ; subject, The Sequelæ of Influenza. A cordial invitation is extended to physicians.

THE *St. Louis Clinique* has passed into the hands of Dr. Emory Lanphear, professor of surgery in the College of Physicians and Surgeons. Dr. Lanphear will conduct the journal in the interests of that school and of the medical profession of the West.

THE *American Journal of Insanity*, which has been edited and published at the Utica State Hospital for the past fifty years, has lately been sold and transferred to the American Medico-Psychological Association, of which society it will henceforth be the accredited organ.

The *Journal* will be edited *ad interim* by a publication committee consisting of Dr. Edward Cowles, president of the association, Boston, Mass.; Dr. Henry M. Hurd, secretary of the association, Johns Hopkins Hospital, Baltimore, Md.; and Dr. Richard Dewey, Chicago, Ill., with the last named gentleman in immediate editorial charge.

Until further notice it will be published in Chicago, Ill.

THE *Doctor and Druggist* is the title of a neat looking little magazine published in St. Louis, the first number of which appeared July 15, 1894. The yearly subscription is $1, and the address is 2622 McNair avenue.

THE *Woman's Medical Journal*, published at Toledo, O., is a magazine that we have a fondness for on account of its high professional and literary tone; but we do wish that it would eliminate its inset advertisements. These neither benefit a journal nor its advertisers, but, on the contrary, often damage both.

THE *Lehigh Valley Medical Magazine* is one of the best edited as well as one of the best printed magazines that comes to our table. The editor very properly refers to the success of the magazine in its last issue, which was the concluding number of its fifth volume. He says the magazine has been forced to make its own standard because among the many medical journals of the country there was not one that could serve as a model. True! and we congratulate the accomplished editor in having achieved a deserved measure of success.

The *Columbus Medical Journal*, published and edited for the last twelve years by Dr. J. F. Baldwin, has passed into the hands of Dr. R. Harvey Reed, and will hereafter be published as a bi-weekly. The first issue under the new *regimé* appeared July 10, 1894, and is a handsome specimen of journalism.

---

The *Maryland Medical Journal* has established a column entitled Current Editorial Comment, in which is given some of the bright thoughts of the contemporary medical press. This is an excellent plan and serves to keep medical journals more in touch with each other as well as with the profession.

---

The *Medical and Surgical Reporter* has a special department entitled Current Literature Reviewed, which is in charge of Drs. Elliston J. Morris and Samuel M. Wilson. In this subdivision of the journal three or four magazines are taken up each week and a summary of their important papers are given. We commend this as an excellent plan.

---

An important new book just announced is Practical Urinalysis and Urinary Diagnosis. A manual for the use of practitioners and students, with numerous illustrations, including colored photo-engravings. By Charles W. Purdy, M. D., of Chicago, author of Bright's Disease and Allied Affections of the Kidneys ; Diabetes : Its Causes, Symptoms and Treatment, etc. A one-volume practical and systematic work of about 350 crown-octavo pages, in two parts, subdivided into twelve sections, and an appendix.

The well-known house of The F. A. Davis Company, 1914 and 1916 Cherry street, Philadelphia, will issue the work in September, 1894. The book will be first-class in quality of paper, press-work and binding, and the price most reasonable—namely, $2.50, net, in extra cloth.

---

The Author of Gallegher.—Philadelphia is Richard Harding Davis's birthplace, writes Edward W. Bok in an interesting sketch, with portrait, of the popular young author in the August *Ladies' Home Journal.* His education began at the age of nine at the Episcopal Academy in that city. In 1882, he became a student at Lehigh University. There he became an enthusiastic football player, and there, too, did his first writing as editor of the college paper. He wrote a dozen stories for the paper, and afterward collected them, put them into a book and paid $90 to get the book published. It had a limited sale—very limited. From Lehigh he went to Johns Hopkins University for a year, and there he wrote, reckoned professionally, his first story, Richard Carr's Baby, a tale with football tendencies. It appeared in *St. Nicholas.*

In 1887, the young student returned to Philadelphia and became a reporter, taking assignments from several newspapers, and earning the princely salary of $7 a week.

Mr. Davis is well, almost magnificently, built, standing six feet high, weighing 180 pounds, and with a physical strength that many envy. He has a frank, boyish manner about him, and is conscious of his success, as is only natural and quite pardonable, but he is not so in any obtrusive way. His manner of talking is quick, his laugh is of the heartiest, and he is an ideal companion. Popular in society, he is sought by everyone and lionized generally. But, with all his success, he is conscious only of a desire to give to the reading public a book or a story that will be superior to the last.

---

Dr. Bransford Lewis has retired from the active editorial management of the *Medical Fortnightly*, but remains president of the Fortnightly Press Company. The journal is now edited by Dr. Frank P. Norbury and Dr. William M. Robertson.—*Medical Bulletin.*

---

Dr. H. C. Minor has retired from the management of the Hot Springs *Medical Journal.* Dr. J. T. Jelks has become associated with the editorial staff and management of the same journal.—*Medical Bulletin.*

